# Correction: Athanasiou, C.-E. et al. A Monolithic Micro-Tensile Tester for Investigating Silicon Dioxide Polymorph Micromechanics, Fabricated and Operated Using a Femtosecond Laser. *Micromachines*, 2015, *6*, 1365–1386

**DOI:** 10.3390/mi9040164

**Published:** 2018-04-02

**Authors:** Christos-Edward Athanasiou, Yves Bellouard

**Affiliations:** Galatea lab, STI/IMT, Ecole Fédérale Polytechnique de Lausanne (EPFL), Rue de la Maladière, 71b, CH-2002 Neuchâtel, Switzerland; yves.bellouard@epfl.ch

The authors would like to make the following changes to the published paper [1]: Equation (5) should be written as follows:

(5) $$\begin{array}{c} F\approx \frac{6wtE_{sio_{2}}\delta^{4}}{3\delta l^{3}-\sqrt{6}wl^{3}coth\sqrt{\frac{3}{2}}\left(\frac{\delta }{w}\right)} \end{array}$$

Equation (13) should be written as follows:

(13) $$\begin{array}{c} u_{\sigma }=\sqrt{{\left(\frac{1}{Ct}\right)}^{2}u_{R}^{2}+{\left(\frac{R}{Ct^{2}}\right)}^{2}u_{t}^{2}} \end{array}$$

The changes do not affect the scientific results. We apologize for any inconvenience caused to the readers by these errors. The manuscript will be updated and the original will remain online on the article webpage, with a reference to this Correction.
